# Stabilized filter-supported bilayer lipid membranes (BLMs) for automated flow monitoring of compounds of clinical, pharmaceutical, environmental and industrial interest

**DOI:** 10.1155/S1463924697000011

**Published:** 1997

**Authors:** Dimitrios P. Nikolelis, Christina G. Siontorou

**Affiliations:** Laboratory of Analytical Chemistry Department of Chemistry University of Athens Panepistimiopolis Athens 15771 Greece

## Abstract

This paper describes the results of analytical applications of electrochemical biosensors based on bilayer lipid membranes (BLMs) for the automated rapid and sensitive flow monitoring of substrates of hydrolytic enzymes, antigens and triazine herbicides. BLMs, composed of mixtures of egg phosphatidylcholine (egg PC) and dipalmitoylphosphatidic acid (DPPA), were supported on ultrafiltration membranes (glass microfibre or polycarbonate filters) which were found to enhance their stability for flow experiments. The proteins (enzymes, antibodies) were incorporated into a floating lipid matrix at an air-electrolyte interface, and then a casting procedure was used to deliver the lipid onto the filter supports for BLM formation. Injections of the analyte were made into flowing streams of the carrier electrolyte solution and a current transient signal was obtained with a magnitude related to the analyte concentration. Substrates of hydrolytic enzyme reactions (acetylcholine, urea and penicillin) could be determined at the micromolar level with a maximum rate of 220 samples/h, whereas antigens (thyroxin) and triazine herbicides (simazine, atrazine and propazine) could be monitored at the nanomolar level in less than 2 min. The time of appearance of the transient response obtained for herbicides was increased to the order of simazine, atrazine and propazine which has permitted analysis of these triazines in mixtures.


Journal of Automatic Chemistry, Vol. 19, No. 1 (January–February 1997) pp. 1–8

### Stabilized filter-supported bilayer lipid membranes (BLMs) for automated flow monitoring of compounds of clinical, pharmaceutical, environmental and industrial interest

**Dimitrios P. Nikolelis* and Christina G. Siontorou**

*Laboratory of Analytical Chemistry, Department of Chemistry, University of Athens, Panepistimiopolis 15771 Athens, Greece*

*This paper describes the results of analytical applications of electrochemical biosensors based on bilayer lipid membranes (BLMs) for the automated rapid and sensitive flow monitoring of substrates of hydrolytic enzymes, antigens and triazine herbicides. BLMs, composed of mixtures of egg phosphatidylcholine (egg PC) and dipalmitoylphosphatidic acid (DPPA), were supported on ultrafiltration membranes (glass microfibre or polycarbonate filters) which were found to enhance their stability for flow experiments. The proteins (enzymes, antibodies) were incorporated into a floating lipid matrix at an air-electrolyte interface, and then a casting procedure was used to deliver the lipid onto the filter supports for BLM formation. Injections of the analyte were made into flowing streams of the carrier electrolyte solution and a current transient signal was obtained with a magnitude related to the analyte concentration. Substrates of hydrolytic enzyme reactions (acetylcholine, urea and penicillin) could be determined at the micromolar level with a maximum rate of 220 samples/h, whereas antigens (thyroxin) and triazine herbicides (simazine, atrazine and propazine) could be monitored at the nanomolar level in less than 2 min. The time of appearance of the transient response obtained for herbicides was increased to the order of simazine, atrazine and propazine which has permitted analysis of these triazines in mixtures.*

### Introduction

Lipid membrane based biosensors have recently been described as a useful tool for monitoring at a continuous or single format mode a large number of compounds of clinical, pharmaceutical, environmental and industrial interest [1–6]. These systems provide a route of generic transduction of an analytical signal with advantages of high sensitivity and selectivity, and fast response times; lipid membranes can also be excellent host matrices for the maintenance of the activity of many biochemically selective species, such as enzymes, antibodies, and nucleic acids [2–4]. Using conductance changes through bilayer lipid membranes (BLMs) containing ion-channel forming species is an important avenue of research for devising chemical sensors. While there have been numerous reports already published on this concept, the main problem has been the lack of a simple/robust experimental arrangement that would enable practical analytical measurements using such principles. Recent research was focused on the preparation of mechanically stabilized BLM assemblies to increase their potential for use as practical biosensors, and to further extend areas of applications. Significant progress in the stabilization of BLMs has been achieved by using ultrafiltration membranes to support BLMs [3–6], and by the use of freshly cut metallic surfaces on which BLMs can form [2, 7–9].

This paper describes the use of BLMs supported on ultrafiltration membranes (glass microfibre or polycarbonate) for the direct continuous monitoring of compounds of clinical, pharmaceutical, environmental and industrial interest. The ultrafiltration systems enhance the stability of BLMs for use in flow injection monitoring of substrates of hydrolytic enzymes [3], antigens (with concurrent regeneration of antibody binding activity) [4] and herbicides [6]. The devices are a cost efficient easy-to-use, fast-responding alternative to standard laboratory analytical and screening methods. Within a single device, a primary transducing element, similar to biological recognition mechanisms, and micromachining technologies are merging to form a unique sensing system. The results from applications of the present thin lipid film technology highlight an improvement of the characteristics of lipid membrane transducers in terms of ruggedness, time of analysis and precision, and these systems offer significant advantages over the existing methods of analysis such as liquid chromatographic (LC) procedures and chromogenic immunoassays.

### Experimental

The lipids used for BLM-based biosensors for flow injection experiments include egg phosphatidylcholine (egg PC) and a charged lipid (used to modulate membrane electrostatics or phase structure) such as dipalmitoylphosphatidic acid (DPPA) [10, 11]. These lipid membranes were formed from dilute lipid solutions (0·04 mg/ml total lipid) and contained 15, 35 and 60% (w/w) DPPA. The solutions were prepared daily from stock solutions of PC (2·5 mg/ml) and DPPA (2·5 mg/ml) in n-hexane-absolute ethanol (80 + 20). The stock lipid solutions were stored in the dark in a nitrogen atmosphere at −4 °C. The filters and (nominal) pore sizes used to support stabilized lipid membranes for flow

*Correspondence to Dimitrios P. Nikolelis.

0142-0453/97 $12·00 © 1997 Taylor & Francis Ltd

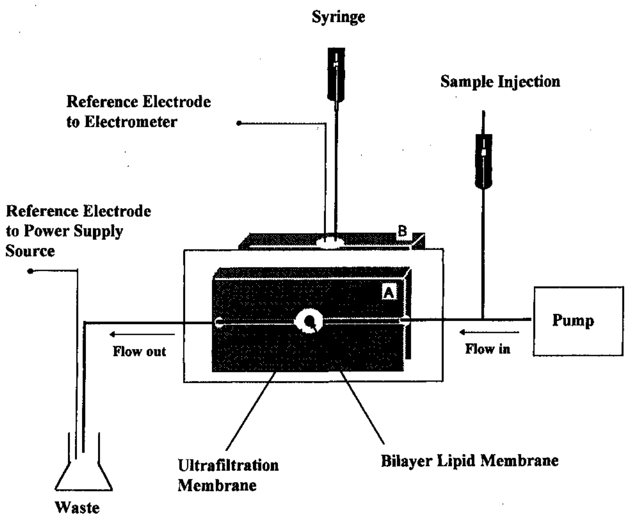

*Figure 1. Simplified schematic of the apparatus used for the formation of the filter supported BLMs for flow through experiments.*

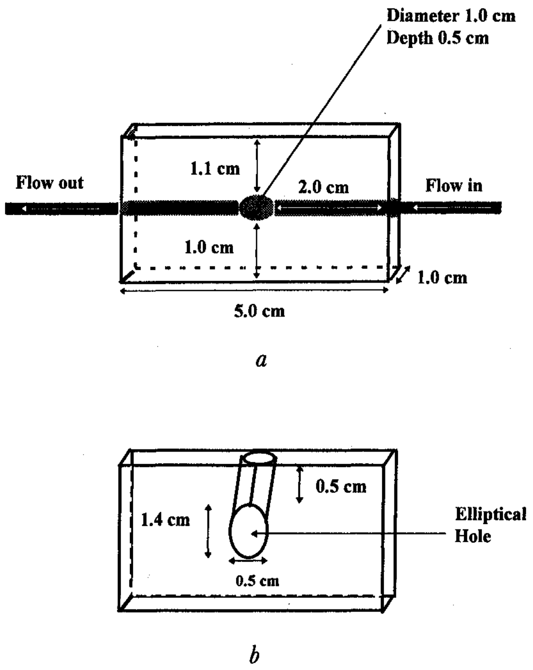

*Figure 2. Electrochemical cells used. (a) Chamber for the flow of the carrier solution. (b) Chamber used for lipid-protein codeposition and casting of the lipid bilayer on the plastic sheet partition.*

injection experiments were GF/F glass microfibre (0·7 μm, Whatmam Scientific Ltd, Kent, UK) and Uni-Pore polycarbonate (1·0 μm, Bio-Rad Laboratories, Mississauga, USA). Other chemicals included gramicidin to characterize the bilayer structure of lipid membranes and HEPES to prepare buffer. The lipid film membranes were supported in a 0·1 M KCl electrolyte solution (buffered with 10 mM HEPES). Water used in experiments was purified by passage through a Milli-Q cartridge filtering system (Milli-Q, Millipore, El Paso, TX, USA) with minimum resistivity of 18 MΩ cm.

The apparatus for the formation of stabilized BLMs for flow injection experiments has been described elsewhere [3–5]; it consists of two identical Plexiglas chambers which are separated by a plastic sheet (PVDC) partition of a thickness of about 10 μm (see figure 1). This PVDC was cut to more than twice the size of the contact area of the faces of the chambers and folded in half; an orifice of 0·32 mm diameter was made through the double layer of the plastic film by punching with a perforation tool [12]. A microporous glass fibre or polycarbonate filter disc (diameter about 9 mm) was placed in this aperture between the two plastic layers, with the filter centred on the 0·32 mm hole. The partition with the filter in place was clamped tightly between the Plexiglas chambers. One of the Plexiglas chambers was machined to contain an electrochemical cell connected with a plastic tubing for the flow of the carrier electrolyte solution (see figure 2[a]); a Ag/AgCl reference electrode was immersed in the waste of the carrier electrolyte solution. The second Plexiglas chamber was machined to contain a cell with a cylindrical shape, having its longitudinal axis perpendicular to the flow of the carrier electrolyte solution of the opposing cell (see figure 2[b]). The upper circular hole of this cell was 1·5 mm from the front and 2·5 mm from the back of the Plexiglas chamber; the lower elliptical hole faced the circular hole of the opposing cell chamber and the 0·32 mm circular aperture was placed approximately at the centre of the assembly. An Ag/AgCl reference electrode was placed into the cylindrical cell and an external 25 mV d.c. voltage was applied across the lipid membrane between the two reference electrodes. A digital electrometer (Model 614, Keithley Instruments, Cleveland, USA) was used as a current-to-voltage converter. A peristaltic pump (Masterflex with SRC Model 7020 speed controller and 7014 pump head) was used to carry the electrolyte solution from the reservoir. Sample injections were made in close proximity to the detector system with a Hamilton repeating dispenser with a disposable tip (Hamilton Co., Nevada, USA). The electrochemical cell and electronic equipment were isolated in grounded Faraday cage.

Stabilized BLMs for flow injection experiments were formed by dropwise addition of a lipid solution (10 μl) using a microlitre syringe to the electrolyte surface in the cylindrical cell (see figure 2[b]) near the partition. The level of the electrolyte solution was dropped below the aperture and then raised again within a few seconds. The formation of a membrane was verified by the magnitude of the ion current obtained, and electrochemical characterization using gramicidin D. Over 95% of attempts (with a freshly prepared dilute lipid solution) for BLM formation were successful and the obtained membranes were stable for periods of more than 8 hours. All experiments were made at 25 ± 1 °C.

### Results and discussion

### *Analytical applications of BLM-based sensors*

Recent studies by the authors were focused on the induction of sensitive response of conventional freely-suspended BLMs to various analytes, investigations of mechanism of signal generation by using differential scanning calorimetry and scanning electron microscopy and strategies to develop stabilized filter- or metal-supported lipid membrane based devices for practical biosensor implementation for monitoring or screening (in a single format) of a wide range of compounds of biomedical, pharmaceutical, environmental and industrial interest [2–6, 8, 9, 13]. The techniques used to prepare stabilized BLM-based sensing devices include the use of filters (glass microfibre and polycarbonate) to support BLMs for flow-through experiments [3–6], preparation of self-assembled BLMs supported on metal electrodes (with long-term stability and constant response characteristics) [8–9] and chemical immobilization of lipids on gold surfaces (suitable for dry wet state cycling experiments). Compounds that can be determined using the techniques developed by the authors include substrates of hydrolytic enzyme reactions (acetylcholine, urea and penicillin) [3], antigens (thyroxin) [4], insecticides (monocrotofos and carbofuran) [13], triazine herbicides (simazine, atrazine, and propazine) [6], gas pollutants (ammonia, and carbon dioxide) [8, 9], and taste substances [14]. The authors have recently published work on the uses of BLMs for the modification of carbon sufaces to develop DNA sensors for the rapid detection of nucleic acids [2].

*Filter-supported BLMs for flow injection monitoring of acetylcholine, urea and penicillin*

Stabilized systems of BLMs composed of egg PC and DPPA and supported on ultrafiltration membranes (polycarbonate and glass microfibre filters) were used for the rapid and sensitive flow injection monitoring of substrates of hydrolytic enzyme reactions (acetylcholine, urea and penicillin) in flowing solution streams [3]. Volumes of 3 µl of solutions of acetylcholinesterase in 10 mM Tris-HCl buffer (pH 7·4) containing 0·4 mg of solid/ml, urease in 50% glycerol and penicillinase (each containing 0·25 mg of solid/ml) are co-deposited with the lipid solution at the air/electrolyte interface prior to BLM formation to maximize the loading of these biological species in the BLMs. The aggregation of charged protein molecules and interactions with the charged lipid component of the BLMs (protein binding to the hydrogen bond accepting sites of DPPA or electrostatic interactions between DPPA and the enzymes [15]) can induce electrostatic field gradients at a BLM surface [16] which would result in restructuring of the BLM double layer. The use of large amounts of proteins causes destabilizing effects of membranes due to concurrent protein-lipid and protein-protein interaction indicating that a critical protein-to-lipid ratio exists, beyond which BLM destabilization and rupture occurs.

The AchE/Ach, urease/urea and penicillinase/penicillin interactions were examined at pH values of 8·0, 6·0 and 7·0, respectively, in the presence of calcium ions as a compromise between optimum enzyme activity and enhancement of signal sensitivity. Solutions of the substrates of the enzymes were injected into flowing streams of a carrier electrolyte solution after the formation of the lipid membrane. Hydronium ions produced by the enzymatic reaction at the BLM surface caused dynamic alterations of the electrostatic fields and phase structure of BLMs and as a result ion current transients were obtained [5]. Figure 3 shows recordings of the signals obtained for different concentrations of Ach; the transient responses appear as singular events as a result of the hydrolytic enzymatic reaction. The magnitude of these transient responses is in direct proportion to the Ach concentration in the carrier electrolyte solution $\{\Delta I\ (pA) = 1{\cdot}419\ [Ach]\ (\mu M) + 2{\cdot}064,\ r^2 = 0{\cdot}9994\}$. The variability of response of the BLMs to the repetitive substrate injections and the ability to reproducibly incorporate active protein is also indicated in figure 3 (RSD 5·1). Similar transient signals were obtained for the penicillinase-catalysed hydrolysis of penicillin, whereas the signals for the urea/urease system were opposite in direction. In general, a good linear correlation was

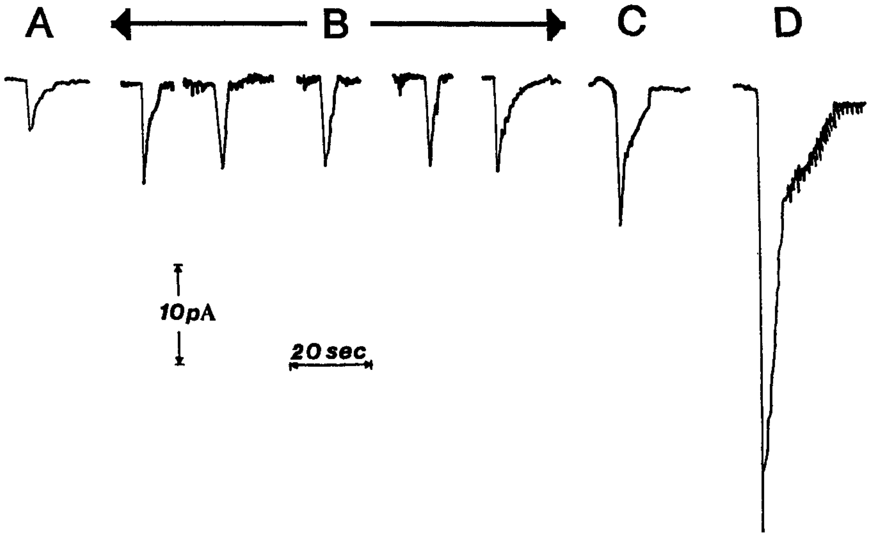

*Figure 3. Experimental results obtained for the AchE/Ach reaction at pH 8·0 (0·1 M KCl, 10 mM HEPES and 1 mM $Ca^{2+}$) with BLMs composed of 35% DPPA and supported in glass microfibre filters. Ach concentrations (mM): (A) 2·00; (B) 5·00; (C) 10·0; (D) 30·0. Samples were injected at the beginning of each recording. The recordings shown in (B) were selected randomly from a large number of injections.*

achieved {ΔI (pA) = 0·231 [Urea] (μM) + 3·868, $r^2$ = 0·990, for urea, and ΔI (pA) = 42·33 [penicillin] (mM) + 1·377, $r^2$ = 0·997 for penicillin} and replicate analyses of substrate samples indicated that the reproducibility is on the order of 5%. The present method offers response times on the order of 10 s, which are the fastest achieved so far as compared to other similar detectors. The detection limits in the present studies are of the order of 1, 10 and 100 μM for Ach, urea and penicillin, respectively, which are similar to those obtained by fluorescence methods. Very good reversibility is also observed with repetitive determinations of substrates in our system with no sample carryover or membrane memory effects (see figure 3). The return to the baseline after each measurement was almost instantaneous, and this permits repetitive determinations of substrates with a rate of 220 samples/hour.

*Filter-supported BLMs as electrochemical detectors for flow immunoanalysis*

A limited number of immunosensors for use in flow injection analysis have recently appeared in the literature [17–22]. However, these immunosensors require the use of acidic [23, 24] or chaotropic media [25] to regenerate the antibody binding sites which permanently alters the antibody conformation or replacement of the chemistry at the device surface, which is costly and time consuming [17–19]. Regeneration of active sites of antibodies without loss of activity has been reported in a few instances by washing with a flowing electrolyte solution (i.e. by mass action) [20–22] which is limited by the kinetics of the antibody-antigen dissociation.

Filter-supported stabilized BLMs composed of egg PC and DPPA were used for the rapid electrochemical monitoring of the Thyroxin (T4)/anti-rabbit T4 reaction in flowing solution streams [4]. The antiserum solution (3 μl of 0·62 mg/ml in phosphate buffer, pH 7·4) was incorporated into a floating lipid matrix at the air/electrolyte interface, and then a casting procedure was used to deliver the lipid onto the filter supports for BLM formation. When the ion current stabilized (over a period of 20 minutes) the antigen solution (75 μl) was injected into the carrier electrolyte solution. The experiments for the calibration graph were performed in a stopped flow mode using 15% (w/w) DPPA to provide only a single transient current signal [26]. Initially, a flow rate of 1·0 ml/min was used, and the flow of the carrier electrolyte solution was stopped 15 s after antigen injection and was again initiated after a single transient signal was obtained (figure 4). The magnitude of the current transients was related to the logarithm of antigen concentration {ΔI (pA) = 14·98 log[Thyroxin] + 127·92, $r^2$ = 0·954}. A 5 min period of washing out with the carrier electrolyte solution was adequate to regenerate the antibody binding sites for multiple repetitive injections of antigen. This period was determined by use of BLMs composed of 35% (w/w) DPPA to provide multiple transient signals [26]. These experiments provided evidence that the time delay between antigen injection and the appearance of the first signal was *c*. 3 min. The flow of the carrier solution was therefore reinitiated after this period of time (i.e. the appearance of the first signal), so that the dissociation of the antibody-antigen complex could be observed as a reduction of the frequency and the magnitude of the transient signals. Figure 5 shows the

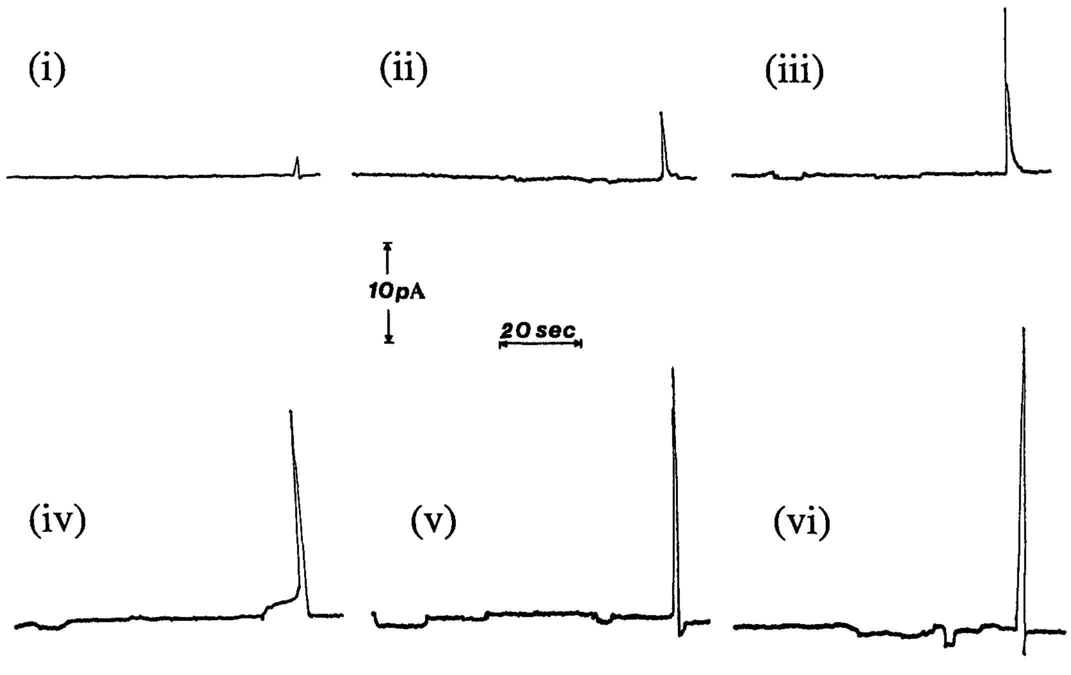

*Figure 4. Experimental results obtained at pH 6·0 (0·1 M KCl, 10 mM HEPES and in the absence of $Ca^{2+}$) with BLMs composed of 15% (w/w) DPPA when 3 μl of antibody stock solution was co-deposited onto the air/electrolyte interface. Thyroxine concentrations (M): (i) $3{\cdot}50 \times 10^{-9}$; (ii) $1{\cdot}00 \times 10^{-8}$; (iii) $3{\cdot}50 \times 10^{-8}$; (iv) $7{\cdot}00 \times 10^{-8}$; (v) $1{\cdot}00 \times 10^{-7}$; (vi) $3{\cdot}50 \times 10^{-7}$. Recordings began when the flow of the carrier solution stopped (15 s after injection of antigen).*

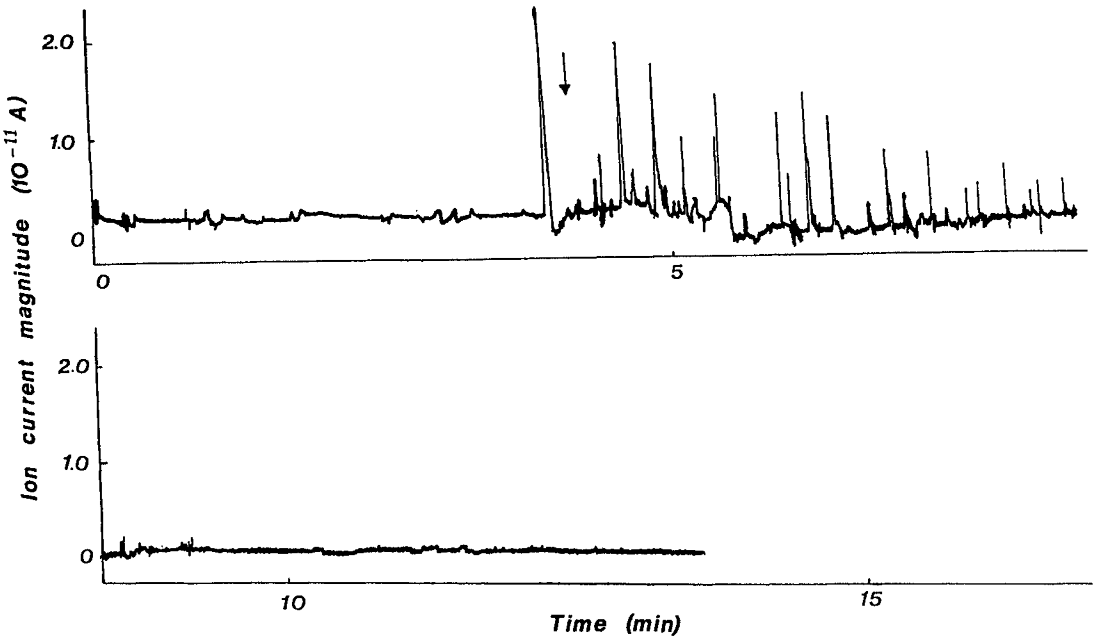

*Figure 5. Recording showing degradation of the first signal observed after the injection of $1 \times 10^{-6}$ M of antigen solution in the carrier electrolyte solution (0·1 M KCl, 10 mM HEPES and 1·0 mM $Ca^{2+}$ at pH 6·0) with membranes consisting of 35% (w/w) DPPA. The flow was initiated again immediately after the appearance of the first signal. The arrow indicates when the flow of carrier solution started.*

reduction, and ultimately the disappearance, of the transient signals obtained by reinitiating the flow of the carrier electrolyte solution after the appearance of the first transient signal (flow reinitiated at time marked by arrow in figure 5). A time of *c*. 5 min was required for the disappearance of the transient signals when using 1 μM of antigen, and decreased for lower concentrations of antigens. Therefore, a lapse of 5 min. was allowed between experiments involving repetitive injections of antigen.

Figure 6 shows the results of a cycle of experiments that used repetitive injections of antigens. The maximum number of injections which could be achieved with retention of calibration of the analytical signal was about five, with a variability of response of the BLMs between 3 and 11% ($N = 5$). The reduction of the signal magnitude with more than five injections was probably due to removal of antibody from BLMs by the flowing solutions. Protein denaturation was not the cause of the loss of the signal, since preparation of the fresh BLMs from the original lipid-protein mixture at the air/electrolyte interface provided full regeneration of antibody binding activity that could be used for another cycle of five injections. The dissociation of antibody from BLMs due to the flow of the carrier electrolyte solution was dependent on the number of injections and on the time that membranes containing antibody were exposed to the flowing solution (flow rate 1·0 ml/min). The signal magnitude decreased after four injections made within 1 hour, or after three injections within 2 hours (all injections were sequential, at equal time intervals).

The results presented in this paper demonstrate the potential of BLMs for applications in flow injection immunoanalysis when using a stopped-flow mode for a limited number of repetitive antigen injections. The results show that stabilized BLMs containing antibody can be used for flow monitoring of antigens at nM levels, and that regeneration of antibody active sites is fast, simple and inexpensive compared to other previously described methods [17–22]. The preparation of BLMs providing full antibody binding activity is fast and simple (a film casting step), and permits preparation of lipid membranes for another cycle of antigen injections without disassembly and cleaning of the detection chamber.

### *Stabilized BLMs for direct electrochemical monitoring of mixtures of simazine, atrazine and propazine*

It was recently suggested that BLMs had the potential of being the basis of devices for the construction of one-shot biosensors for direct monitoring insecticides [13]; the suggestion was to use planar 'free-suspended' BLMs. However, the adaptation of the interactions of insecticides with BLMs using filter-supported BLMs for the continuous flow monitoring of insecticides is limited by the time delay of insecticide adsorption and partitioning into the lipid membrane and the mechanism of signal generation. This is because insecticides are believed to be buried deeply into BLMs [27]. In an effort to find environmental pollutants which could potentially be monitored using the stabilized systems of BLMs supported in microporous filters [5] (in a continuous or stopped-flow mode), the interactions of triazine herbicides (atrazine, simazine and propazine) with stabilized BLMs composed of egg PC and DPPA were examined [6]. The injections of the herbicide solutions were made into flowing streams of a carrier electrolyte solution and transient current signals with a duration of seconds reproducibly appeared in less than 2 min after exposure of the lipid membranes to the herbicides.

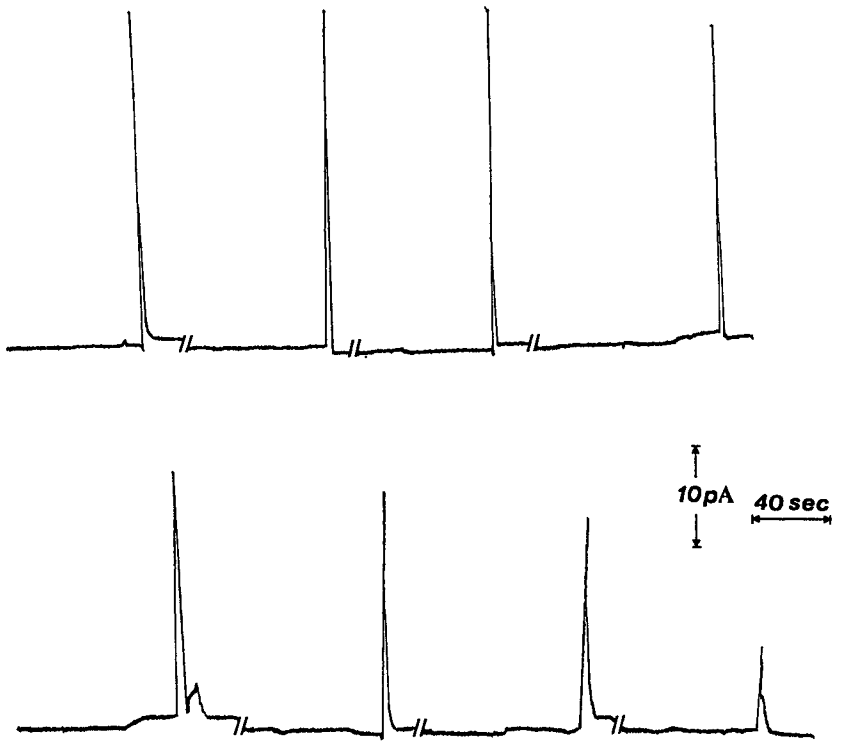

*Figure 6. Signals obtained during a cycle of repetitive injections of 3·50 × $10^{-7}$ M T4 at pH 6·0 (0·1 M KCl, 10 mM HEPES and in the absence of $Ca^{2+}$) with BLMs composed of 15% (w/w) DPPA when 3 μl of solution containing 0·62 mg/ml antibody was co-deposited onto the air/electrolyte interface. The time delay between the repetitive injections (marked as // in the figure) was 5 min.*

Figure 7 shows recordings of the signals obtained with injections of atrazine in continuous flowing streams of carrier electrolyte solution of pH 8·0 (0·1 M KCl, 10 mM HEPES, and 1·0 mM $Ca^{2+}$) and flow rate 1·1 ml/min. A transient current signal as a single event was obtained by the interactions of atrazine with the filter-supported BLMs. A constant time delay for the appearance of the transient currents of 70·5 ± 6·5 s can be seen in figure 7. The magnitude of these signals was linearly related to atrazine concentration, which could be determined at sub-micromolar level {$\Delta$I (pA) = 35·5 [atrazine] (ppm) + 1·46, $r^2$ = 0·994}. The current transients observed when simazine and propazine were injected to the flowing stream of the electrolyte solution (i.e. also noticed as singular events) were similar to those of atrazine, and their magnitude could be used to quantify the concentrations of simazine and propazine in the carrier electrolyte solution.

Statistical treatment of results gave regression equations: $\Delta$I (pA) = 0·295 [simazine] (ppb) + 0·226, $r^2$ = 0·997 for simazine, and $\Delta$I (pA) = 0·157 [propazine] (ppb) + 3·78, $r^2$ = 0·999 for propazine. In general, a good linear correlation was observed and replicate analyses of simazine and propazine samples indicated that the reproducibility was less than 6%. The calibration graph for the triazine herbicides determination has shown linearity in the concentration range between 0·050 to 1·4 ppm for atrazine, 18 to 210 ppb for simazine, 30 to 300 ppb for propazine. The calibration graph declines for higher concentrations of triazines [28]. The detection limits in the present study are in the order of 40, 8, 20 ppb for atrazine, simazine and propazine, respectively. Repetitive cycles of injection of herbicides have shown no signal degradation during each cycle, implying that there is no sample carryover or membrane memory effects (the relative standard deviation was 2·9%). The return to the baseline after each measurement was almost instantaneous, and this permits repetitive triazine determinations with a rate of about one sample/min.

The time of appearance of the transient response due to BLM/herbicide interactions was different for each triazine and increased to the order of simazine, atrazine and propazine which has allowed selective detection and analysis of these triazines in mixtures. The time delay for the appearance of these transients were 40·7 ± 5·1 s for simazine and 118 ± 14·4 s for propazine. The range of time delay for the appearance of the transient signals was between 34 to 50 s for simazine ($\mathcal{N}$ = 11), 62 to 78 s for atrazine ($\mathcal{N}$ = 11) and 96 to 144 s for propazine ($\mathcal{N}$ = 11). The differences observed in the delay time for the appearance of signal of these herbicides has allowed the determination of simazine, atrazine and propazine in mixtures. Figure 8 shows recordings obtained for such mixtures containing variable amounts of these herbicides. Figure 8 shows that a discrete signal is obtained for each herbicide in mixture, and not an integral response of BLMs (i.e. a single transient corresponding to the overall effect of these herbicides in mixture). The resolution of the peaks of each triazine obtained in such mixtures was sufficient, thus allowing reliable selective monitoring of the herbicides in mixture. The recovery of the triazine herbicides in mixture was

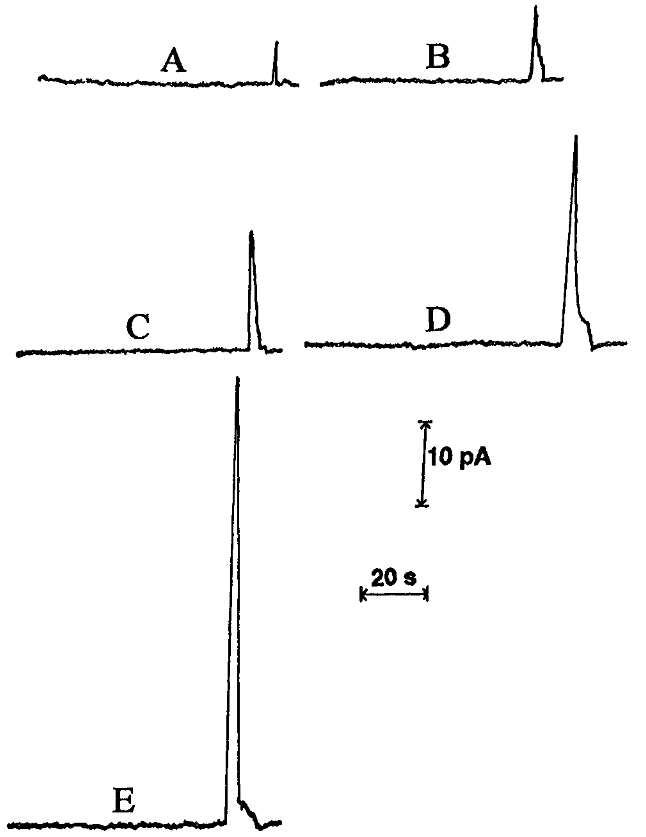

*Figure 7. Signals obtained for atrazine at pH 8·0 (0·1 M KCl, 10 mM HEPES and 1·0 mM $Ca^{2+}$) with BLMs composed of 35% (w/w) DPPA and supported in glass microfibre filters. Atrazine concentrations of the solutions injected (75 μl) in the carrier electrolyte (ppm): (A) 0·0875; (B) 0·175; (C) 0·350; (D) 0·700; (E) 1·40. Samples were injected at the beginning of each recording.*

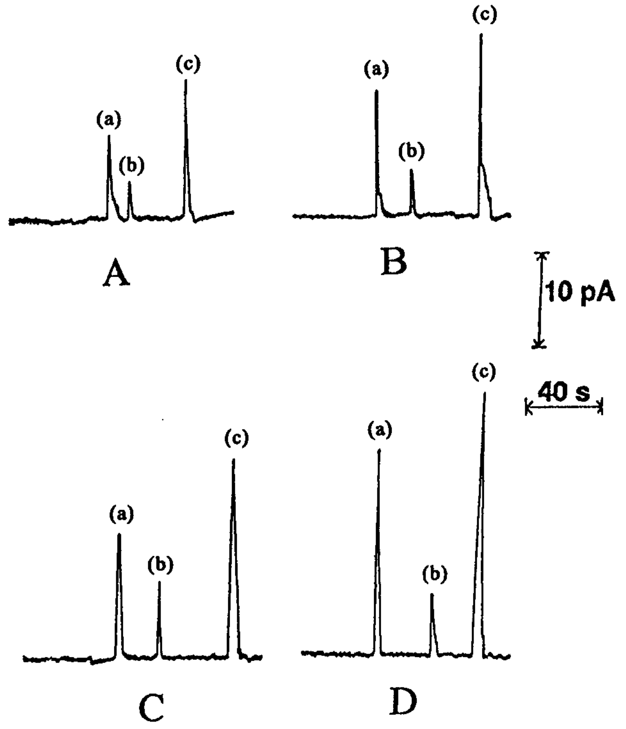

*Figure 8. Signals obtained for mixtures of simazine (a), atrazine (b) and propazine (c) using the experimental conditions described in figure 7. The solutions injected into the carrier electrolyte contained: (A) simazine 30·0 ppb; atrazine 70·0 ppb; 66·7 ppb. (B) simazine 44·4 ppb; atrazine 91·6 ppb; propazine 100 ppb. (C) simazine 44·4 ppb; atrazine 183 ppb; propazine 100 ppb. (D) simazine 75·0 ppb; atrazine 133 ppb; propazine 150 ppb. Samples were injected at the beginning of each recording.*

complete. However, analysis of mixtures of triazines containing total amounts of more than *c.* 500 ppb demonstrated that the recovery of atrazine was satisfactory, whereas the recovery of simazine and propazine produced negative errors (i.e. a mixture containing amounts of 175 ppb of each triazine has provided peaks corresponding to 84·0, 185 and 69·0 ppb for simazine, atrazine and propazine, respectively). These negative errors for simazine and propazine are due to the limitations previously described for the linearity of calibration graphs and relevant saturation of the BLM.

The present BLM-based system is able to monitor triazine herbicide in mixtures and offers response times of the order of less than 2 min., which are the fastest achieved up to now as compared to other similar detectors. The present method offers a simultaneous and repetitive mode of detection of triazine herbicides, which is faster and has a lower cost than that based on antibodies or photosynthetic systems [18, 29]. The results of the flow injection analysis of triazine mixtures using the BLM-based detection scheme are analogous to the resolution of the peaks obtained using chromatographic procedures. The direct flow monitoring and injection analysis of these environmental pollutants should be applied in protein-free water samples, as proteins or small peptides may cause a non-selective interference with BLMs [8]. A number of pesticides and insecticides were tested as potential interferents; the compounds tested were diuron ($1{\cdot}8 \times 10^{-4}$ M), alachlor ($3{\cdot}7 \times 10^{-5}$ M), chloropyrifos ($5{\cdot}7 \times 10^{-6}$ M), carbofuran ($4{\cdot}5 \times 10^{-4}$), monocrotofos ($1 \times 10^{-4}$ M), aldicarb ($3{\cdot}2 \times 10^{-5}$ M) and methylparathion ($1{\cdot}9 \times 10^{-4}$ M). These compounds did not cause any transient signals at the concentration levels shown in parentheses; this is probably due to the faster adsorption times of triazine herbicides as compared to the above potent interferents [13]. The interactions of the triazine herbicides with BLMs and the obtained electrochemical signals are specific in the presence of other coexisting compounds; the latter generally require membrane modification by incorporation of 'receptor' (i.e. enzyme, antibody or receptor) molecules into the lipid matrix for the purpose of obtaining an analytical signal. The results in this paper show that it is possible to induce specificity of BLM-based devices to discriminate herbicides in the presence of insecticides or pesticides. The technique described has higher detection limits than those obtained by chromatographic methods; however, it has significant advantages over these procedures, for example analysis times, sample volumes, as well as the size and cost of chromatographic instrumentation.

*Effect of flow rate*

The filter supported BLMs described in this paper are more suitable for practical biosensor implementation, such as flow through applications, than the conventional freely suspended BLMs [12]. However, noise level substantially increases with an increase in flow rate and typical flow rates that can be used are up to 3·2 ml/min, which result in noise levels of less than 1 pA. The signal magnitude and delay time of signal appearance decreases as the flow rate increases when substrates of hydrolytic enzyme reactions are monitored. The delay time was decreased from about 6 s to 3 s when the flow rate increased from 1·7 ml/min to 3·2 ml/min, but the signal magnitude was decreased to 60% as observed with injections of Ach in the BLM-acetylocolinestarase system.

Further experiments were performed to exploit the effect of the carrier flow rate on the number of antigen injections that could be done in each cycle of injections when using the filter supported BLMs in flow immunoanalysis. A flow rate of 2·0 ml/min was found to limit the number of injections which could provide a calibrated transient signal to two. These results are consistent with the hypothesis of antibody dissociation from membranes previously described.

The effect of the flow rate on signal magnitude is not very critical when the interaction of triazine herbicides with BLMs are monitored when using the filter-supported BLM systems. A signal of constant magnitude is obtained when using flow rates up to *c.* 2 ml/min and the signal decreases for flow rates higher than 2 ml/min. These results provide evidence that the adsorption of triazines in BLMs is not the rate-determining step in the mechanism of signal generation for flow rates between 0 to 2 ml/min.

### Conclusions

The results from applications of the present thin lipid film technology show that microfabricated stabilized BLM-based biosensors for flow injection analysis provide fast response times (of the order of seconds), high sensitivity, submicromolar detection limits, reversibility, capability of analysing small volumes of samples, and can now be reliably fabricated with simplicity and low cost for the development of automated methods for the determination of compounds of biomedical, pharmaceutical, environmental and industrial interest. An improvement of the characteristics of lipid membrane transducers has been achieved by supporting BLMs in ultrafiltration membranes for uses in flow injection experiments in terms of ruggedness, time of analysis and precision. The technique has significant advantages over the existing methods of analysis, such as LC procedures (i.e. analysis times, size and cost of LC instrumentation limit the use of this technology for screening applications in the field) [30] and chromogenic immunoassays (which are highly sensitive and selective, but they take from many minutes to hours to complete and usually require multiple steps, both of which hamper their adaptation to biosensor format) [31]. Work is in progress to extend the versatility of choice of a wide range of chemically-selective reagents, and is focusing on the use of these stabilized filter supported BLMs for the development of an automated method for the determination of aflatoxin and other toxins in foodstuffs and the construction of DNA sensors for the rapid monitoring environmental pollutants and carcinogens.

### Acknowledgements

This work was carried out as part of the 'Copernicus' project, Contract no. CIPA CT94-0231 with a financial contribution from the European Commission.

### References

1. Nikolelis, D. P. and Krull, U. J., *Electroanalysis*, **5** (1993), 539.

2. Siontorou, C. G., Brett, A.-M. O. and Nikolelis, D. P., *Talanta*, **43** (1996), 1137.

3. Nikolelis, D. P. and Siontorou, C. G., *Analytical Chemistry*, **67** (1995), 936.

4. Nikolelis, D. P., Siontorou, C. G., Andreou, V. G., Viras, K. G. and Krull, U. J., *Electroanalysis*, **7** (1995), 1082.

5. Nikolelis, D. P., Siontorou, C. G., Andreou, V. G. and Krull, U. J., *Electroanalysis*, **7** (1995), 531.

6. Nikolelis, D. P. and Siontorou, C. G., *Electroanalysis*, **18** (1996), 907.

7. Tien, H. T. and Salamon, Z., *Bioelectrochemistry & Bioenerg.*, **22** (1898), 211.

8. Nikolelis, D. P., Siontorou, C. G., Krull, U. J. and Katrivanos, P. L., *Analytical Chemistry*, **68** (1996), 1735.

9. Nikolelis, D. P. and Siontorou, C. G., *Bioelectrochemistry & Bioenerg.*, in press.

10. Nikolelis, D. P., Brennan, J. D., Brown, R. S. and Krull, U. J., *Analytica Chimica Acta*, **257** (1991), 49.

11. Nikolelis, D. P. and Krull, U. J., *Analytica Chimica Acta*, **257** (1992), 239.

12. Nikolelis, D. P. and Krull, U. J., *Talanta*, **39** (1992), 1045.

13. Nikolelis, D. P. and Krull, U. J., *Analytica Chimica Acta*, **288** (1994), 187.

14. Cucu, D., Mihailescu, D., Mihailescu, G., Nikolelis, D. P., Flonta, M.-L. and Frangopol, P. T., *Biophysical Chemistry*, in press.

15. Boggs, J. M., *Biochemical Cell Biology*, **64** (1986), 50.

16. Lundstrom, I., *FEBS Letters*, **83** (1977), 7.

17. Bier, F. F. and Schmid, R. D., *Biosensors & Bioelectronics*, **9** (1994), 125.

18. Bier, F. F., Jockers, R. and Schmid, R. D., *Analyst*, **119** (1994), 437.

19. Pollema, C. H. and Ruzicka, J., *Analytical Chemistry*, **66** (1994), 1825.

20. Locascio-Brown, L., Plant, A. L., Horvath, V. and Durst, R. A., *Analytical Chemistry*, **62** (1990), 2587.

21. Yap, W. T., Locascio-Brown, L., Plant, A. L., Choquette, S. J., Horvath, V. and Durst, R. A., *Analytical Chemistry*, **63** (1991), 2007.

22. Locascio-Brown, L., Plant, A. L., Chesler, R., Kroll, M., Ruddel, M. and Durst, R. A., *Clinical Chemistry*, **39** (1993), 386.

23. Sutherland, R. M., Dahne, C., Place, J. F. and Ringrose, A. S., *Clinical Chemistry*, **30** (1984), 1533.

24. Tromberg, B. J., Sepaniak, M. J., Vo-Dinh, T. and Griffin, G. D., *Analytical Chemistry*, **59** (1987), 1226.

25. Bright, F. V., Betts, T. A. and Litwiler, K. S., *Analytical Chemistry*, **62** (1990), 1065.

26. Nikolelis, D. P., Tzanelis, M. G. and Krull, U. J., *Analytica Chimica Acta*, **282** (1993), 527.

27. Balke, N. E. and Price, T. P., *Pesticide Biochemistry & Physiology*, **30** (1988), 228.

28. Nikolelis, D. P. and Andreou, V. G., *Electroanalysis*, **18** (1996), 643.

29. Wortberg, M., Middendorf, A., Katenkamp, A., Rump, T., Krause, J. and Camman, K., *Analytica Chimica Acta*, **289** (1994), 177.

30. Analytical currents, *Analytical Chemistry*, **66** (1994), 569A.

31. Analytical currents, *Analytical Chemistry*, **66** (1994), 81A.

